# The gut microbiome participates in transgenerational inheritance of low‐temperature responses in *Drosophila melanogaster*

**DOI:** 10.1002/1873-3468.13278

**Published:** 2018-11-15

**Authors:** Aman Zare, Anna‐Mia Johansson, Edvin Karlsson, Nicolas Delhomme, Per Stenberg

**Affiliations:** ^1^ Department of Molecular Biology Umeå University Sweden; ^2^ Division of CBRN Security and Defence FOI‐Swedish Defence Research Agency Umeå Sweden; ^3^ Department of Forest Genetics and Plant Physiology Umeå Plant Science Centre Swedish University of Agricultural Sciences Umeå Sweden; ^4^ Department of Ecology and Environmental Science (EMG) Umeå University Sweden

**Keywords:** *Drosophila melanogaster*, environmental response, gut microbiota, host–microbiome interaction

## Abstract

Environmental perturbations induce transcriptional changes, some of which may be inherited even in the absence of the initial stimulus. Previous studies have focused on transfers through the germline although microbiota is also passed on to the offspring. Thus, we inspected the involvement of the gut microbiome in transgenerational inheritance of environmental exposures in *Drosophila melanogaster*. We grew flies in the cold versus control temperatures and compared their transcriptional patterns in both conditions as well as in their offspring. F2 flies grew in control temperature, while we controlled their microbiota acquisition from either F1 sets. Transcriptional status of some genes was conserved transgenerationally, and a subset of these genes, mainly expressed in the gut, was transcriptionally dependent on the acquired microbiome.

## Abbreviation

**OPLS‐DA**, orthogonal projections to latent structures discriminant analyses

**OTU**, operational taxonomic unit

**PCA**, principal component analysis

**TMM**, trimmed mean M‐values

It is widely accepted that organisms adapt to changes in the environment mainly through changes in gene expression patterns, which frequently include both general and specific stress responses 1. Traditionally, it was believed that such changes have no direct effect on transcription patterns of subsequent generations. However, it is now known that responses to environmental changes can be transmitted to subsequent generations. This phenomenon can occur even if the offspring are not exposed as germ cells or any later life stages to the environmental conditions that induced changes in their parents, grandparents or great‐grandparents 2. Moreover, responses to various types of changes, e.g. dietary alteration or diverse types of stress, appear to persist through several generations and can be passed through both maternal and paternal lines 3, 4. The mechanisms responsible for such transmission of responses over several generations are debated. Some studies suggest that small RNAs (coding and non‐coding) are involved in some cases 5, 6, 7. However, germ cells are frequently not the only cells that are transferred to the next generation. Much of the microbiota associated with many multicellular organisms, such as the *Drosophila melanogaster* considered here, are also transferred to the next generation 8, 9.

In recent years, it has been progressively established that the microbiota, especially the gut microbiota, plays essential roles in animals’ viability and health 10. Microbial or faecal transfer from healthy donors is also a promising treatment for various diseases 11, 12, 13. Strikingly, there are examples of unwanted phenotypes being transferred with the microbiota in faecal transfer experiments. For instance, a woman with recurrent *Clostridium difficile* infection reportedly developed new‐onset obesity after receiving microbiota from a healthy but overweight donor 14.

Taken together, this evidence indicates that the microbiome may mediate some of the transgenerational inheritance of responses to environmental changes. Eggs (and to some extent sperm) can transfer such memory from one generation to the next as many RNAs and proteins are deposited in gametes. In the study presented here, we investigated whether microbiota may also be involved in transgenerational transfers of responses to environmental changes.

## Methods

### Fly handling

*Drosophila melanogaster* isogenic flies (*w* ^*1118*^_*iso*_ */Dp(1;Y)y* ^*+*^_*iso*_ *;2*_*iso*_ *;3*_*iso*_) were reared on potato mash‐yeast‐agar medium in plastic bottles and kept at 25 °C until the amount of parental flies needed for the experimental procedure was reached. For the food preparation, 5.5 L tap water was brought to a boil. 200 g Instant mashed potato, 50 g Agar, 250 mL Corn Syrup and 80 g Dry yeast were added under vigorous stirring. The food was boiled for 15 min and then allowed to cool down to 60 °C before adding 42 mL Nipagin (100 g·L^−1^) and 5.5 g Ascorbic acid. About 50 mL food was dispensed into each bottle under constant stirring.

Four biological replicates each were done for both the cold condition (18 °C) and control temperature (25 °C) and the flies were kept in a humidified incubator on a 12‐h light/12‐h dark cycle. To promote egg‐laying of F1 embryos, the parental flies were transferred twice to new bottles at 48‐h intervals before being discarded. Flies of the F1 generation were allowed to lay eggs on apple juice plates at their cultivation temperature (18 or 25 °C). Apple juice plates were made by dissolving 27 g agar in 1 L ultrapure water. The solution was autoclaved and sucrose (33 g) and apple juice concentrate (330 mL) were added. Once the temperature reached 60 °C, 20 mL Nipagin (100 g·L^−1^) was added and the solution poured into 60 mm Petri dishes (10 mL medium per Petri dish). Embryos from each replicate were collected every third hour, washed once with Embryo wash solution (0.4% NaCl, 0.02% Triton X‐100), once with 2% Na‐hypochlorite (for 10 s), once again with Embryo wash solution and finally once with ultrapure water before being transferred to bottles containing faeces from F1 males cultivated at either 18 or 25 °C. To supplement the fly food with the microbiome of the previous generation, F1 males grown at 18 or 25 °C were placed in new bottles at 18 or 25 °C respectively, where they lived for 2 days to transfer their gut microbiota. After removal of the F1 males, the washed eggs originating from 18 or 25 °C were transferred to the bottles containing F1 faeces in all four possible combinations. All F2 generation flies were then cultivated in a humidified incubator at 25 °C with 12‐h light/12‐h dark cycles.

### RNA sequencing

#### RNA preparation

For RNA sequencing, 20 adult males (0–16 h old) from each biological replicate of each category of F1 and F2 flies were collected. The flies were snap‐frozen in liquid nitrogen and stored at −80 °C. RNA was isolated with TRI Reagent according to the manufacturer's instructions (Thermofisher, Waltham, MA, USA), treated with Baseline‐Zero DNase (Epicentre, Madison, WI, USA) for 15 min at 37 °C and purified using an RNeasy MinElute Cleanup Kit (Qiagen, Hilden, Germany). To deplete ribosomal RNA, the samples were treated with Ribo‐Zero Epidemiology (Illumina, San Diego, CA, USA) according to the manufacturer's instructions. The Ribo‐Zero‐treated RNA was purified using a modified RNeasy MinElute Cleanup Kit (Qiagen), according to the TruSeq Stranded mRNA Sample Preparation Guide (Illumina).

#### Library preparation

RNA libraries were prepared using an Illumina TruSeq^®^ Stranded mRNA LT Sample Prep Kit. The first step of the standard protocol (polyA purification) was skipped to collect the total RNA. Five microlitre of each prepared RNA solution was transferred to a 96‐well PCR plate and mixed with 13 μL of Fragment, Prime, Finish Mix and the resulting mixture was incubated at 94 °C for 8 min. The libraries were subsequently prepared according to Illumina′s protocol, from the next step, ‘Synthesize First Strand cDNA’, onwards.

#### Sequencing

The samples were sequenced using an Illumina HiSeq2500 Platform in multiplexed pools of nine samples per lane. Fragments of a 500 bp average insert size were sequenced paired‐end, for 125 cycles.

### Amplicon sequencing

#### DNA preparation

DNA was prepared from food samples taken before (16 bottles with fresh food) and after microbiome transfer (eight samples from 18 °C and 8 from 25 °C) as well as from five males (0–16 h old) from each of the four replicates from the two F1 conditions and the four F2 conditions. Each sample was homogenized in 465 μL of Bacteria Lysis Buffer (20 mm Tris/HCl pH 8.0, 2 mm EDTA, 1.2% Triton X‐100 and 20 mg·mL^−1^ lysozyme) and incubated for 1.5 h at 37 °C. A 25 μL portion of SDS (10%) and 10 μL proteinase K solution (20 mg·mL^−1^) were added and the resulting mixture was incubated at 55 °C for a further hour. The DNA was isolated by phenol/chloroform extraction followed by ethanol precipitation.

#### Amplicon generation and sequencing

16S rRNA V3‐V4 (bacterial) amplicons were generated by PCR according to the 16S Metagenomic Sequencing Library Preparation guide (15044223B, Illumina), except that 2.5 μL of the solutions of extracted DNA was subjected to 30 amplification cycles (see Table S2 for primer sequences). Resulting concentrations of amplicons were measured by Qubit Fluorometric Quantification (Invitrogen, Carlsbad, CA, USA), and those with a final concentration exceeding 4 nm were pooled at equimolar ratios, while those with lower concentrations were pooled by volume. The pooled amplicons were spiked with 5% PhiX (Illumina) and sequenced using an Illumina Miseq platform and MiSeq reagent kit v3 (paired‐end, 2 × 300 bp).

### Preprocessing of RNA‐Seq data

RNA‐Seq data were processed following published guidelines 15, with some modifications. Briefly, fastqc v0.11.5 16 was used for initial QC assessments of paired‐end reads in FastQ format. Residual ribosomal RNA (rRNA) contamination was assessed and filtered using sortmerna v1.9 17 and the rRNA sequences provided with sortmerna: rfam‐5s‐database‐id98.fasta; rfam‐5.8s‐database‐id98.fasta; silva‐arc‐16s‐database‐id95.fasta; silva‐bac‐16s‐database‐id85.fasta; silva‐euk‐18s‐database‐id95.fasta; silva‐arc‐23s‐database‐id98.fasta; silva‐bac‐23s‐database‐id98.fasta; silva‐euk‐28s‐database‐id98.fasta. fastqc was used again for monitoring the quality of the reads after rRNA sorting. Next, trimmomatic v0.32 18 (using SLIDINGWINDOW:5:20 MINLEN:50 and the TruSeq3‐PE‐2.fa adapter file as part of the Trimmomatic archive) was used with default parameters to remove adapter sequences and low‐quality bases from the reads. Again, fastqc was used to verify the retained reads’ quality and the absence of technical artefacts. Following these quality assessments, multiqc v0.6 19 was used to combine the fastqc results in a single report. Filtered reads were aligned to the *Drosophila melanogaster* reference genome release 6 (dmel‐all‐chromosome‐r6.11.fasta, retrieved from ftp://ftp.flybase.net/genomes/Drosophila_melanogaster/dmel_r6.11_FB2016_03/fasta/) using star 20 (v 2.4.0f1, with non‐default parameters: alignIntronMax 11000 –outSAMstrandField intronMotif –readFilesCommand zcat –outSAMmapqUnique 254 –quantMode TranscriptomeSAM –outFilterMultimapNmax 100 –outReadsUnmapped Fastx –chimSegmentMin 1 –outSAMtype BAM SortedByCoordinate –outWigType bedGraph). Annotations retrieved from FlyBase (dmel‐all‐r6.11.gff) were used to generate synthetic gene models. The synthetic transcript files and alignments from star were used as input for htseq‐count (in the htseq python framework v0.6.1 21) to calculate read counts while taking into account only uniquely mapped reads (non‐default parameters: ‐r pos ‐m intersection‐nonempty ‐s reverse).

### 16S rRNA amplicon sequencing data processing and OTU picking

The reads obtained from sequencing were merged using flash v1.2.11 22 with a minimum overlap of 10 bp and maximum overlap of 300 bp resulting in > 99% of the merged reads to have an overlap of ≥ 85 bp. The merged reads were trimmed using trimmomatic v0.32 with default settings and minimum length set to 350 bp. Greengenes 23 (release 8.15.13) was used as reference sequence database. Before Operational Taxonomic Unit (OTU) picking, an optional prefiltering step (using the Greengenes database) was performed to remove sequences that are not originating from the targeted marker gene or due to sequencing errors (flag: –prefilter_percent_id 60). This and the following steps were done using qiime v1.9.1 24. Open reference OTU picking was then performed, with a 97% sequence identity threshold, using pick_open_reference_otus.py with uclust v1.2.22 25 and the resulting OTUs were filtered for singletons using filter_otus_from_otu_table.py script. Preprocessed OTUs were then rarefied, and alpha diversity metrics were computed using alpha_rarefaction.py workflow script. Finally, beta_diversity_through_plots.py was used for beta diversity assessment (parameters: ‐e 10000) and producing principal coordinate analysis (PCoA) plots using emperor v0.9.51 26.

### RNA‐Seq data analysis

Differential expression of genes between samples of various categories (mentioned above) was analysed using the Bioconductor package edger v3.18.1 27 in r v3.4.0 28. A 0.05 FDR cut‐off was used to select differentially expressed genes. Prior to differential expression computation, the read counts were normalized using trimmed mean M‐values (TMM) 29 in the edger package. The resulting TMM‐normalized matrix was then used for identifying differentially expressed genes and both multivariate and hierarchical clustering analyses. In r, phyper function was used to test the significance of overlapping differentially expressed genes in F1 and F2 generations (shown in Fig. 2A). simca v.14 (Sartorius Stedim Data Analytics AB, Umeå, Sweden) was used for principal component analysis (PCA) and orthogonal projections to latent structures discriminant analyses (OPLS‐DA). For the OPLS‐DA, four models were produced. In each model, one of the replicates for each of the conditions was removed prior to making the model. In the first model, all A‐replicates were removed, in the second model, all B‐replicates removed, and so on. The excluded A‐replicates were predicted back into the model from which they were previously excluded (the same for B, C and D replicates). Hierarchical clustering was done in r using hclust and pvclust v2.0‐0 30 packages. The distance matrix was calculated using Spearman distances followed by clustering using ward.D as the clustering metric. After bootstrapping with pvclust (nboots = 1000), branches with an AU percentage lower than 75% were collapsed. GO analysis was done using the Panther online tool 31, 32 (Gene Ontology database release 2017‐08‐14), and enrichments were summarized using Revigo online 33. Tissue expression profiles were generated by first extracting the expression data for candidate genes from FlyAtlas (retrieved from flyatlas.org). Then, tissues where each gene is expressed (expression values of 100 or more in FlyAtlas) were extracted and used for summarization into word maps using the free online tool Wordle (www.wordle.net) (see Appendix S2).

## Results and Discussion

**Figure 1:**
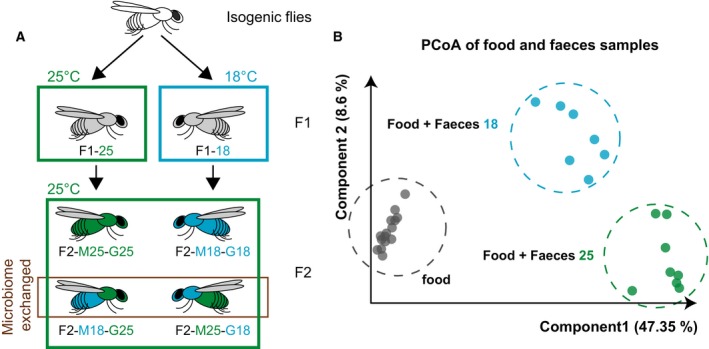
Experimental design to assess generational microbiome transfer. (A) Schematic design of the experiment. Briefly, F2 flies were hatched from washed eggs placed on clean media containing parental microbiota. Colours of the heads and abdominal parts indicate germline and microbiome sources, respectively (blue, cold‐treated flies; green, control flies). Coloured rectangles show the temperature at which the flies were grown. (B) Clustering of food samples before and after adding the faeces. PCoA scatterplot is showing distributions of the samples, based on 16S rRNA amplicon sequencing, along with the first two components. The food samples lacking a pre‐incubation with faeces are indicated in grey and samples contaminated with faeces from cold‐treated and control F1 flies are indicated in blue and green respectively.

To study the effects of microbiota transference to offspring, we must be sure to control the microbiome that offspring receive and ensure that the experimental design allows exchanges of microbiomes between individuals. For this, *D. melanogaster* is a good model system as fruit flies directly lay their eggs in their food, where the parents are also defecating. In this study, two sets of F1 flies descended from isogenic flies that had been grown at the standard laboratory temperature of 25 °C, were put in two different temperatures. One set was maintained at a ‘cold’ temperature (18 °C) for their whole life, while the other set was kept at 25 °C. These sets are hereafter referred to as cold‐treated and control flies. Eggs harvested from both sets were carefully washed and placed in previously boiled food, in which either cold‐treated or control male flies had defecated, thereby ensuring the controlled transfer of specific microbiomes and preventing uncontrolled egg‐laying. To check that the washing (that include a 2% Na‐hypochlorite treatment for 10 s) did not damage the embryos, we counted the number of embryos that lost their dorsal appendages and compared these numbers to unwashed embryos and embryos subjected to a dechorionation protocol (5 min, 3% Na‐hypochlorite treatment). About 2.2% of the untreated embryos (*n* = 726), 3.5% of the washed embryos (*n* = 1505) and 98.1% of the dechorionated embryos (*n* = 1114) had lost their dorsal appendages showing that our washing procedure did not severely damage the chorion. The design of the experiment is schematically illustrated in Fig. 1A. To verify that the males transferred their gut microbial community to the food and to investigate whether the treatment altered its microbial composition, we analysed the food samples before and after defecation by 16S rRNA amplicon sequencing 34. This clearly separated food samples with and without faeces, and food samples with faeces of control and cold‐treated flies (Fig. 1B, for bacterial compositions see Table S3). These findings indicate that bacterial communities with distinct composition were transferred through the faeces of control and cold‐treated flies.

**Figure 2:**
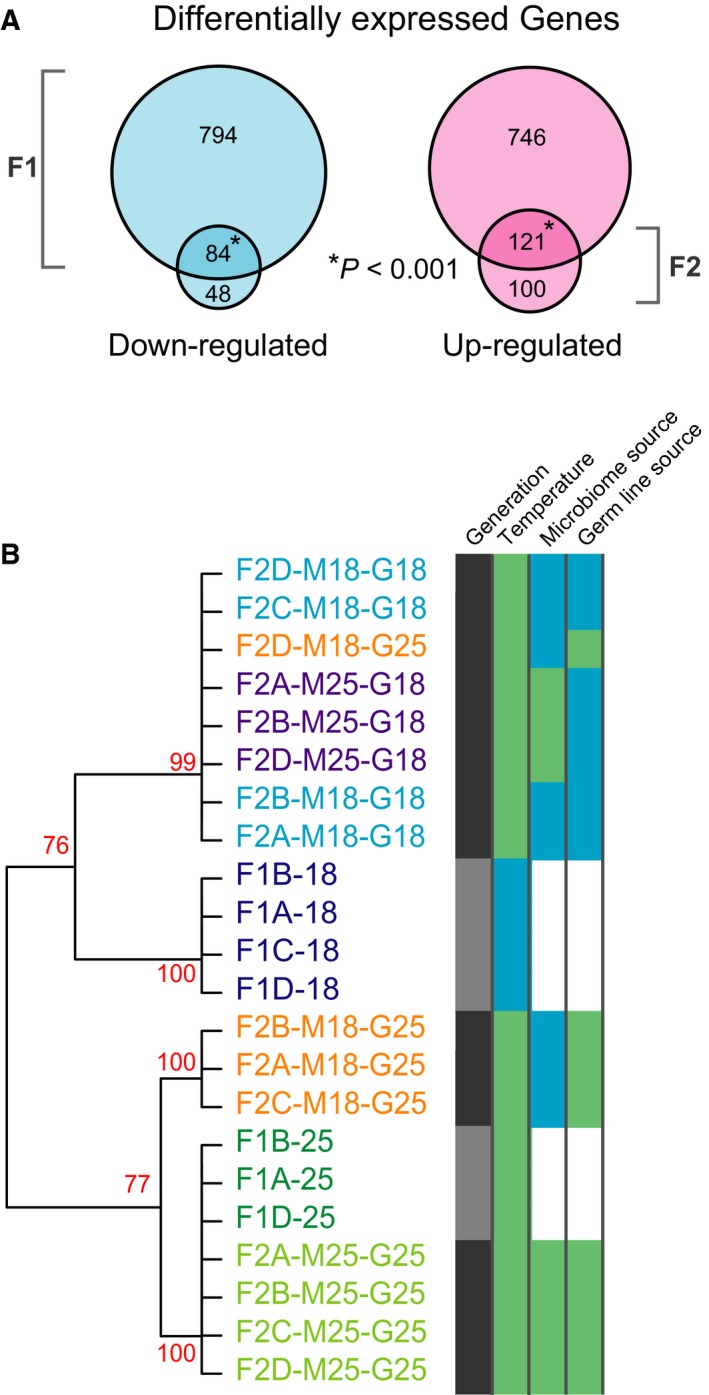
Differentially expressed genes in treated flies and their offspring. (A) Overlaps of differentially expressed genes in the two generations. *P*‐values calculated by hypergeometric test using the number of differentially expressed genes in two generations and the total number of expressed genes in any condition as the reference set (*n* = 12 021). (B) Hierarchical clustering of all samples based on the normalized read count data. Branches with less than 75% AU (coloured red) were collapsed. The colours to the right of the dendrogram indicate replicates’ generation (light grey F1, dark grey F2), cultivation temperature (green 25 °C, blue 18 °C), and F2 flies’ sources of microbiome and germline (F1 flies grown at 25 °C and 18 °C, green and blue respectively).

To study transgenerational temperature responses, the F2 offspring of both cold‐treated and control F1 flies were grown at 25 °C, regardless of the microbiome they acquired through faecal transfer. Males (0–16 h old) were harvested from both generations, and their RNA was extracted for sequencing. After quality filtering and excluding two samples that had <2 million mapped reads, we obtained on average 4.2 million mapped reads per sample (see Table S1). As expected, many genes were differentially expressed between F1 control and cold‐treated flies 35 (for details, see the volcano plot and summary of enriched GO‐terms of the differentially expressed genes in Fig. S1). Moreover, some genes were differentially expressed between F2 offspring that received both germline and microbiome from F1 cold‐treated flies, and F2 offspring that received germline and microbiome from F1 control flies (F2‐M18‐G18 vs F2‐M25‐G25 in Fig. 1A). There is a significant overlap of these genes with differentially expressed genes obtained from the comparison between cold‐treated and control F1 flies (Fig. 2A, *P* < 0.001). Thus, some of the transcriptional responses were clearly retained transgenerationally.

Hierarchical clustering of the transcriptional profiles of all samples showed that the F2 samples clustered primarily according to the source of their germline (with one exception) and together with the F1 samples that provided their germlines (Fig. 2B). Based on these observations, we conclude that most of the transgenerational retention of transcriptional responses was transferred through the germline. Samples of cold‐treated F1 flies form a distinct cluster, but these flies are also the only flies that were not grown in control condition. Interestingly, samples of three (of four) of the F2 flies that received their germline from control flies but microbiome from cold‐treated flies formed a separate sub‐cluster (see Fig. 2B, lower branch). This and the position of another replicate of this group (F2D‐M18‐G25; Fig. 2B) indicates that both the germline and acquired microbiome influenced the F2 flies’ transcriptional patterns.

**Figure 3:**
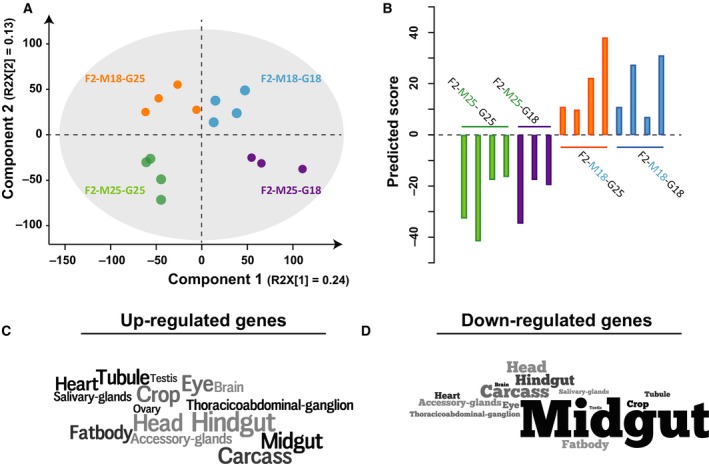
The microbiome significantly contributes to the inheritance of cold acclimation and most of the host–microbiome interaction occurs in the gut. (A) PCA clustering of all offspring based on normalized expression data. The first two components and corresponding *R* ^2^ *X* values in parenthesis are shown. (B) OPLS‐DA predictions of sources of the microbiome in offspring samples. Summary of four models where one replicate of each category was excluded from each model and then predicted back into the model. Each bar shows the predicted score for the replicate removed before modelling. (C) and (D) Enrichment of tissues where the genes whose expression patterns were inherited through the microbiome are expressed (data from FlyAtlas). The size of each word represents the number of these 116 genes (45 up‐regulated and 71 down‐regulated) expressed in each tissue.

It is not surprising that much of the retained transcriptional response was linked to the germline, partly because the F2 offspring were subjected to the cold‐temperature (or control) treatment as eggs. However, the goal of this study was to investigate whether the microbiome also plays a role and to analyse further its effect, we subjected the F2 samples’ expression profiles to a PCA. We found that the first principal component separated F2 flies that received their germline from cold‐treated flies from those that received their germline from control flies (Fig. 3A). Furthermore, flies that acquired their microbiomes from cold‐treated and control flies were separated along the second component. We also subjected the F2 samples to multiple OPLS‐DA using a classifier indicating the source of their microbiomes (cold‐treated or control flies). In each model, we excluded one replicate representing each condition (so there were four models with four removals in each run). All models produced a clear separation of the samples based on their source of the microbiome (positive values in the predictive component for one set and negative for the other). Each model was then used to predict the class (microbiome source) of each of the previously excluded replicates. Excluded replicates were always predicted to belong to the correct class, according to the sign of the predicted value (Fig. 3B). Altogether, we believe that our results unambiguously show, for the first time, that the microbiome can transmit transcriptional responses to environmental changes from one generation to the next. Further study is required to determine whether this phenomenon persists in further generations and, if so, for how many generations.

The microbiome composition of the faeces was clearly distinct between flies reared at 18 °C vs 25 °C (see Table S3). In line with previous studies of lab grown flies, the faecal bacterial composition was dominated by the two families Acetobacteriaceae and Lactobacillaceae 36, 37 where the former is in higher proportion at 18 °C. At 25 °C the proportions of these two families are more similar (Table S3). To investigate whether this composition was stable and maintained also in the F2 flies we performed 16S rRNA amplicon sequencing of adult F2 males from the four conditions. Although, there is a relatively large variation between the replicates, it appears that the F2 flies that got their microbiome from parents reared at 18 °C maintained a higher proportion of Acetobacteriaceae compared to Lactobacillaceae (Table S4 and Fig. S2). On the other hand, the F2 flies that got a 25 °C microbiome maintained similar proportions of the two bacterial families. Future studies will have to determine if the bacterial composition or physiological changes within the bacteria are responsible for transmitting transcriptional responses to environmental changes in the flies, from one generation to the next. Reintroduction of controlled compositions of bacteria into axenic flies could reveal the influence of individual bacterial species on this transgenerational effect. However, producing axenic or xenobiotic flies requires quite harsh treatments, such as broad‐spectrum antibiotics and/or complete dechorionation of the eggs, which will lead to physiological and transcriptional effects in the flies 38, 39, 40. In this study, we made the effort to be as gentle as possible with the flies in order to minimize unrelated effects.

To further investigate the fly genes for which a transcriptional response was explicitly inherited through the microbiome, we selected those that were differentially expressed between cold‐treated and control F1 flies, and which expression status was transmitted to the next generation (overlapping genes in Fig. 2A). From these, we extracted genes (116) that were differentially expressed (in the same direction) between offspring with the same germline but different microbiome sources and determined where these genes are preferentially expressed using flyatlas 41. Of these 116 genes, 45 were up‐regulated in response to cold‐temperature and seemed to be expressed in various tissue types (Fig. 3C, Fig. S2 and Appendix S1). However, most of the 71 genes that were down‐regulated in response to the cold temperature are mainly highly expressed in various parts of the fly's gut (Fig. 3D, Fig. S3 and Appendix S2). This could indicate that a part of the interplay between the microbiome and the fly resulting in transgenerational retention of expression profiles occurs in the gut. However, some of the transcriptional effects observed in our study could be caused by factors not controlled for in the experiment and therefore be microbiome independent. For example, the washing of the eggs might have introduced a transcriptional response. On the other hand, the fact that the F2‐M25‐G25 flies do not cluster separately from the F1‐25 flies in our cluster analysis would indicate that this effect is minor.

The biological importance of transgenerational inheritance of past experiences is hotly debated, and more studies with different model systems are needed. We only investigated the influence of the microbiome over one generation, but strongly believe that our results justify serious consideration of hosted microorganisms in future analyses of transgenerational inheritance of environmental exposures.

## Author contributions

AZ, PS and A‐MJ conceived and designed the experiments; A‐MJ performed all the experiments except the amplicon sequencing which was performed by EK; AZ analysed the data under supervision of PS and ND; AZ and PS wrote the paper.

## Supporting information


**Fig. S1.** Differentially expressed genes when comparing cold‐treated flies versus control (F1).
**Fig. S2.** Tissue expression profile for differentially expressed genes whose expression pattern was inherited through the microbiome.
**Fig. S3.** Relative bacterial composition in the food supplemented with parental faeces, and in the corresponding F2 flies.
**Table S1.** A summary of samples’ read counts.
**Table S2.** Amplicon primers (5′‐3′) used for generating amplicons for 16S V3‐V4.
**Table S3.** Summary of microbial composition and abundance as well as average DNA content in different food conditions with and without addition of faeces.
**Table S4.** Summary of microbial composition and abundance within flies growing on food supplemented with different faeces.
**Appendix S1.** Differentially expressed genes in F1 and F2.
**Appendix S2.** FlyAtlas expression data and the tissue selection for genes used in Figs 3C,D and S2.

## Data Availability

All data sets generated during the current study are available from Gene Expression Omnibus database under accession number GSE111117.
