# Supplementary material for: The gut microbiome participates in transgenerational inheritance of low‐temperature responses in Drosophila melanogaster
Source: FEBS Lett. 2018 Nov 15;592(24):4078–86. doi: 10.1002/1873-3468.13278 (PMC6587461; doi:10.1002/1873-3468.13278)
Supplement: Supplementary file 1 — Fig. S1. Differentially expressed genes when comparing cold‐treated flies versus control (F1). Fig. S2. Tissue expression profile for differentially expressed genes whose expression pattern was inherited through the microbiome. Fig. S3. Relative bacterial composition in the food supplemented with parental faeces, and in the corresponding F2 flies. Table S1. A summary of samples’ read counts. Table S2. Amplicon primers (5′‐3′) used for generating amplicons for 16S V3‐V4. Table S3. Summary of microbial composition and abundance as well as average DNA content in different food conditions with and without addition of faeces. Table S4. Summary of microbial composition and abundance within flies growing on food supplemented with different faeces. Appendix S1. Differentially expressed genes in F1 and F2. Appendix S2. FlyAtlas expression data and the tissue selection for genes used in Figs 3C,D and S2. [file FEB2-592-4078-s001.docx]

**Supplementary Figures**

**
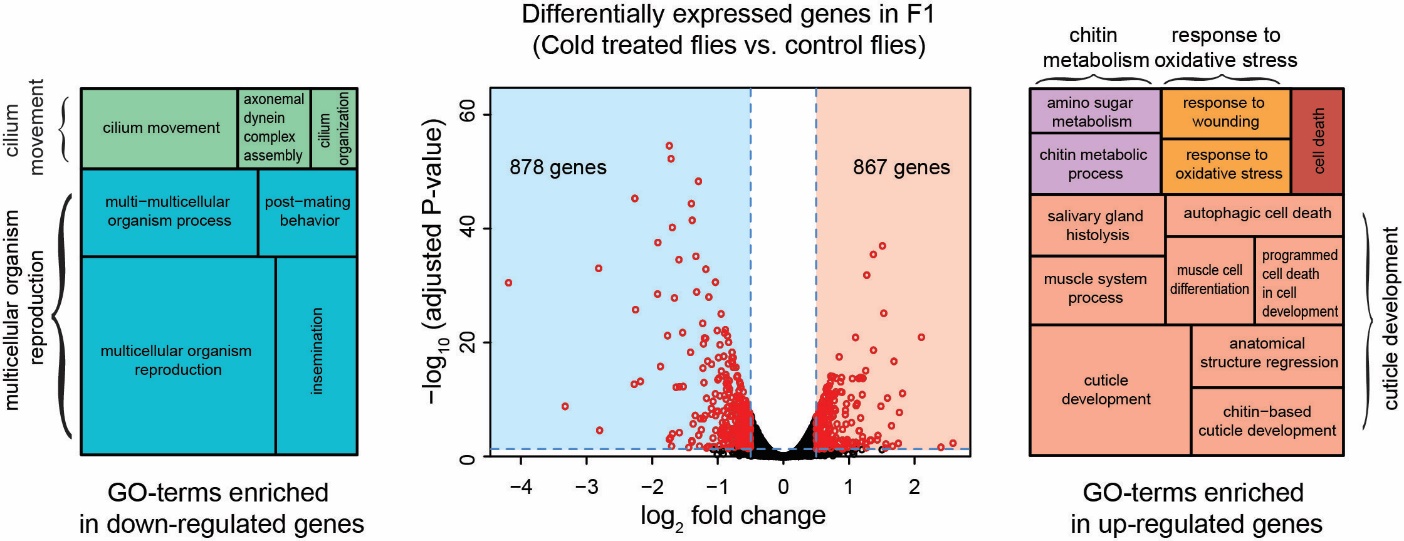
**

**Fig. S1.** Differentially expressed genes when comparing cold treated flies versus control (F1). The Revigo summary of corresponding GO terms (biological processes from Panther) for up and down regulated genes are shown to the sides of the volcano plot.

**
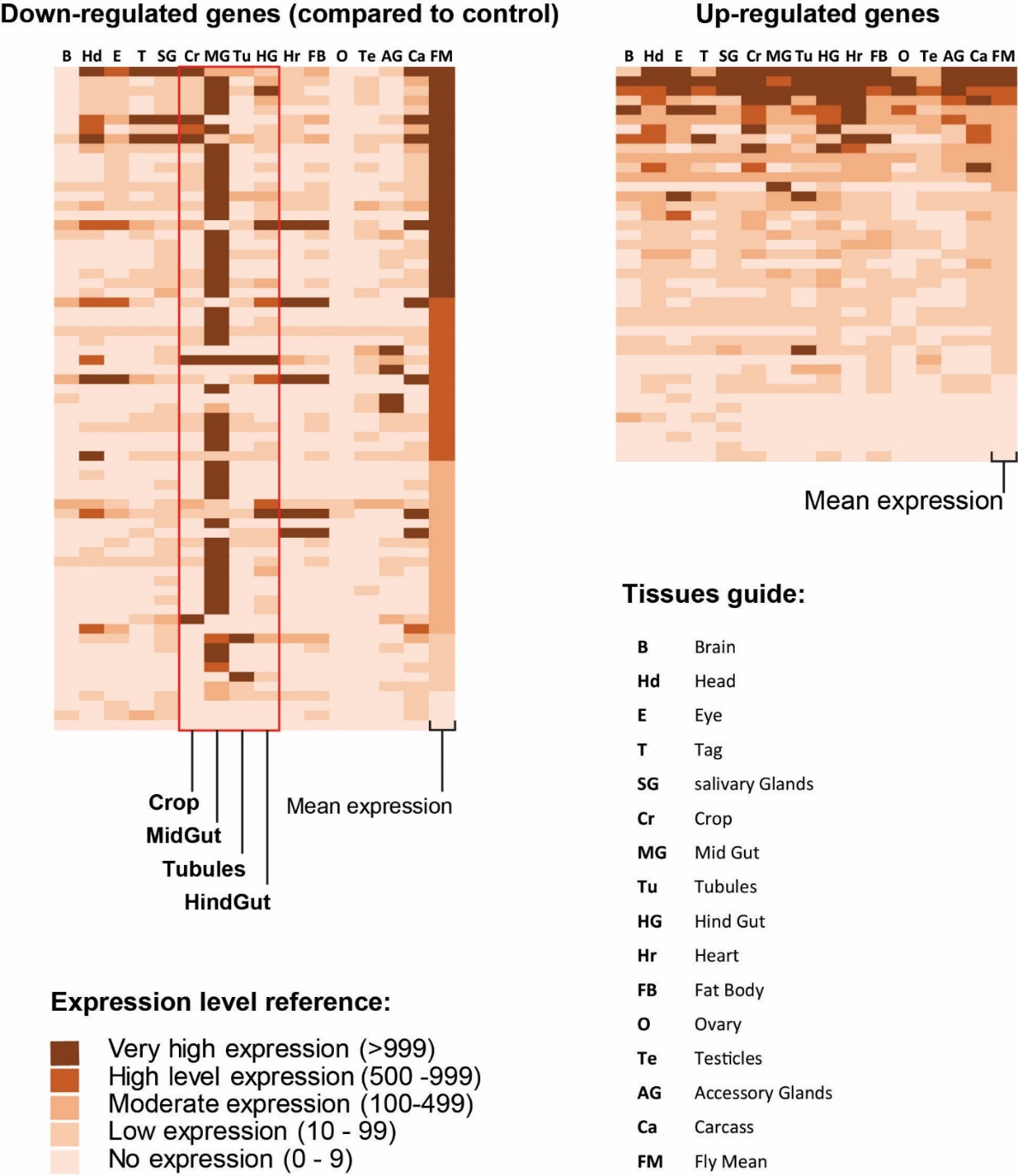
**

**Fig. S2.** Tissue expression profile for differentially expressed genes whose expression pattern was inherited through the microbiome. The heatmaps are showing the expression profiles for fly tissues obtained from Flyatlas. The genes are sorted by the average expression of all tissues (last column in each heatmap). Different parts of the gut are highlighted with a red rectangle in down‐regulated genes (left panel).


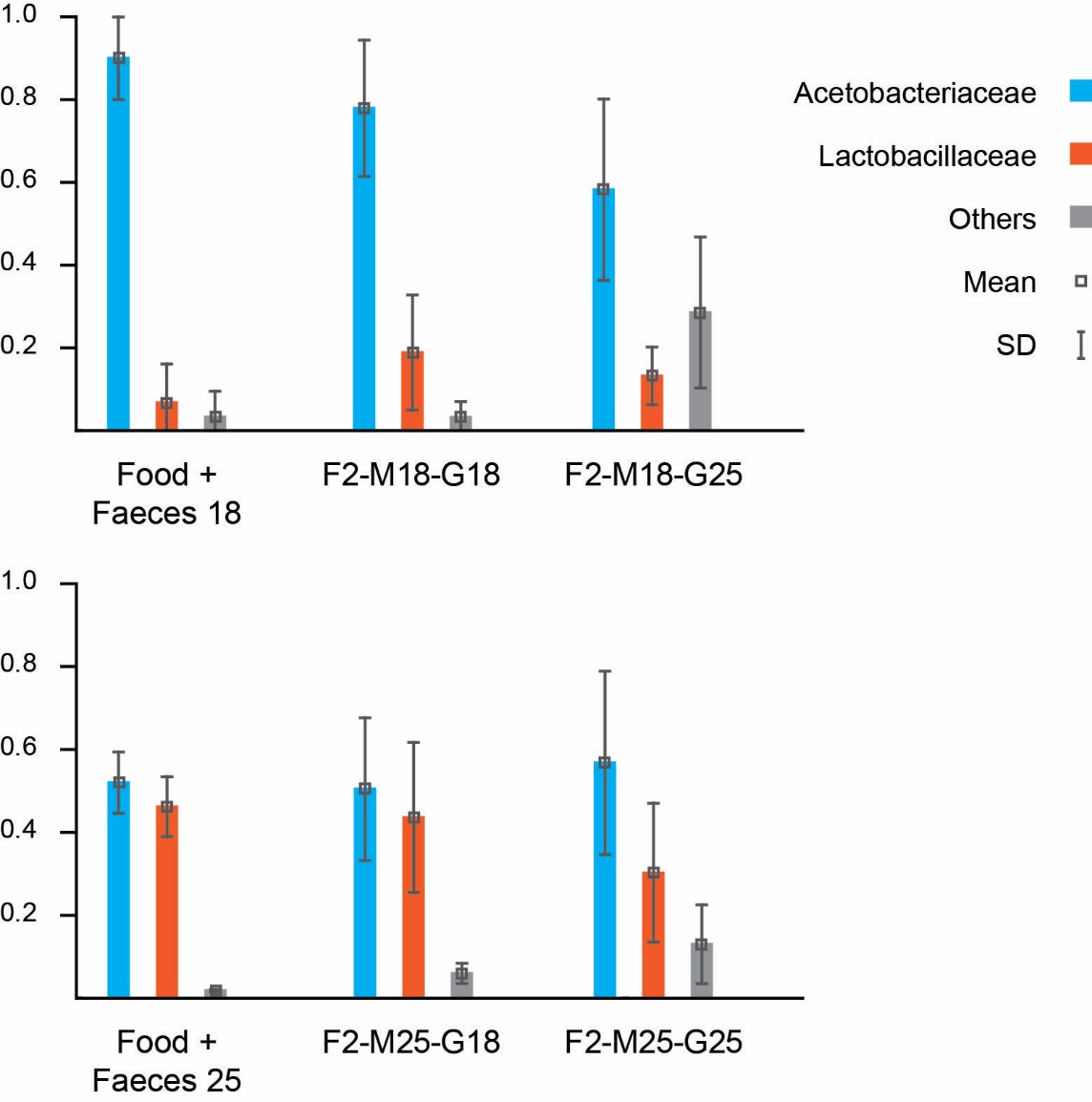


**Fig. S3.** Relative bacterial composition in the food supplemented with parental faeces, and in the corresponding F2 flies. The relative composition of two major bacterial families in the food samples supplemented with the faeces from F1-18 (upper) and F1-25 (lower) is presented in bar plots with mean ± SD.

**Supplementary tables**

**Table S1:** A summary of samples’ read counts

| **Samples** | No feature | Ambiguous | Alignment not unique | **Aligned reads** |
| --- | --- | --- | --- | --- |
| **F1A‐18** | 380185 | 107161 | 2268058 | **5266260** |
| **F1B‐18** | 424396 | 71806 | 1831599 | **3409493** |
| **F1C‐18** | 601815 | 90445 | 2552308 | **4255142** |
| **F1D‐18** | 691638 | 110567 | 2905546 | **5128803** |
| **F1A‐25** | 297113 | 45739 | 1279228 | **2149112** |
| **F1B‐25** | 485713 | 87782 | 2170587 | **4235104** |
| **F1D‐25** | 809845 | 138631 | 3573335 | **6955996** |
| **F2A‐M18‐G18** | 158460 | 30054 | 661221 | **1460861** |
| **F2B‐M18‐G18** | 1102381 | 159898 | 4566738 | **7207240** |
| **F2C‐M18‐G18** | 1156724 | 200147 | 5012891 | **9291907** |
| **F2D‐M18‐G18** | 279221 | 57448 | 1306934 | **2852174** |
| **F2A‐M18‐G25** | 665944 | 117863 | 2847052 | **5645674** |
| **F2B‐M18‐G25** | 302781 | 62390 | 1471394 | **3095597** |
| **F2C‐M18‐G25** | 69940 | 12282 | 281768 | **597427** |
| **F2D‐M18‐G25** | 544482 | 94667 | 2237185 | **4467447** |
| **F2A‐M25‐G25** | 687073 | 128707 | 2968122 | **6243874** |
| **F2B‐M25‐G25** | 338189 | 49965 | 1202675 | **2661568** |
| **F2C‐M25‐G25** | 563491 | 89718 | 2431610 | **4522272** |
| **F2D‐M25‐G25** | 431717 | 64033 | 1669374 | **2945792** |
| **F2A‐M25‐G18** | 466315 | 69970 | 1910008 | **3324271** |
| **F2B‐M25‐G18** | 299254 | 52531 | 1255427 | **2475899** |
| **F2D‐M25‐G18** | 456678 | 69152 | 1804957 | **3629093** |

**Table S2:** Amplicon primers (5’‐3’) used for generating amplicons for 16S V3‐V4.

| **Primer** | **Sequence** |
| --- | --- |
| 16S V3‐V4 Forward | TCGTCGGCAGCGTCAGATGTGTATAAGAGACAGCCTACGGGNGGCWGCAG |
| 16S V3‐V4 Reverse | GTCTCGTGGGCTCGGAGATGTGTATAAGAGACAGGACTACHVGGGTATCTAATCC |

**Table S3:** Summary of microbial composition and abundance as well as average DNA content in different food conditions with and without addition of faeces.

|  | DNA Content (ng/µl) | bacterial Families | Number of reads | Fraction of all reads |
| --- | --- | --- | --- | --- |
| Food | 0.107  (SD=0.085) | Propionibacteriacea | 171642 | 0.248 |
|  |  | Acetobacteriaceae | 111115 | 0.16 |
|  |  | Streptococcaceae | 93536 | 0.135 |
|  |  | Neisseriaceae | 41501 | 0.06 |
|  |  | Staphylococcaceae | 23212 | 0.034 |
|  |  | Corynebacteriaceae | 18724 | 0.027 |
|  |  | Lactobacillaceae | 18584 | 0.027 |
|  |  | Micrococcaceae | 15776 | 0.023 |
|  |  | Others | 213630 | 0.308 |
|  |  | Sum | 691944 | 1 |
| Food + faeces 18 °C | 0.624  (SD=0.299) | Acetobacteriaceae | 1241454 | 0.883 |
|  |  | Lactobacillaceae | 120998 | 0.086 |
|  |  | Others | 43437 | 0.031 |
|  |  | Sum | 1405889 | 1 |
| Food +  faeces 25 °C | 5.104  (SD=2.219) | Acetobacteriaceae | 708405 | 0.518 |
|  |  | Lactobacillaceae | 634041 | 0.463 |
|  |  | Others | 25702 | 0.019 |
|  |  | Sum | 1368148 | 1 |

**Table S4:** Summary of microbial composition and abundance within flies growing on food supplemented with different faeces.

|  | bacterial Families | Number of reads | Fraction of all reads | |
| --- | --- | --- | --- | --- |
| F2-18-18  *(Descendants of F1-18 with microbiome also from F1-18)* | Acetobacteriaceae | 50777 | | 0.862 |
|  | Lactobacillaceae | 7337 | | 0.124 |
|  | Others | 818 | | 0.014 |
|  | Sum | 58932 | | 1 |
| F2-18-25  *(Descendants of F1-25 with microbiome from F1-18)* | Acetobacteriaceae | 13597 | | 0.814 |
|  | Lactobacillaceae | 1120 | | 0.067 |
|  | Propionibacteriaceae | 386 | | 0.023 |
|  | Others | 1601 | | 0.096 |
|  | Sum | 16704 | | 1 |
| F2-25-18  *(Descendants of F1-18 with microbiome from F1-25)* | Acetobacteriaceae | 9717 | | 0.524 |
|  | Lactobacillaceae | 7759 | | 0.418 |
|  | Propionibacteriaceae | 209 | | 0.011 |
|  | Others | 873 | | 0.047 |
|  | Sum | 18558 | | 1 |
| F2-25-25  *(Descendants of F1-25 with microbiome also from F1-25)* | Acetobacteriaceae | 6628 | | 0.562 |
|  | Lactobacillaceae | 3852 | | 0.327 |
|  | Propionibacteriaceae | 122 | | 0.01 |
|  | Others | 1183 | | 0.1 |
|  | Sum | 11785 | | 1 |
